# Advantages of DES over BMS in Preventing the Risk of Myocardial Infarction, Ischemic Stroke, and Mortality in Various Populations

**DOI:** 10.3390/jcm12010024

**Published:** 2022-12-20

**Authors:** Pei-Ning Wu, Jia-Hung Chen, Chuan-Pin Yang, Jason C. Hsu

**Affiliations:** 1 National Health Insurance Administration-Southern Division, Ministry of Health and Welfare, Tainan 700203, Taiwan; 2 Research Center of Data Science on Healthcare Industry, College of Management, Taipei Medical University, Taipei 110301, Taiwan; 3 International Ph.D. Program in Biotech and Healthcare Management, College of Management, Taipei Medical University, Taipei 110301, Taiwan; 4 Clinical Data Center, Office of Data Science, Taipei Medical University, Taipei 106339, Taiwan; 5 Clinical Big Data Research Center, Taipei Medical University Hospital, Taipei Medical University, Taipei 110301, Taiwan

**Keywords:** heart–brain axis, drug-eluting stent, bare-metal stent, ischemic stroke, prevention

## Abstract

**Backgrounds:** Previous studies have demonstrated that drug-eluting stents (DESs) are more effective than bare metal stents (BMSs) in reducing the risk of myocardial infarction in the short term, but the long-term preventive benefits for myocardial infarction, ischemic stroke, and mortality are not clear. **Objective:** This study deeply analyzed the long-term (within 3 years) advantages of the use of DESs in preventing the risk of myocardial infarction, ischemic stroke, and mortality in various populations compared with those of using BMSs. **Methods:** This was a retrospective observational cohort study. We used the 2015–2019 claims data from Taiwan’s National Health Insurance Research Database. Patients over the age of 18 who underwent coronary stent placement (both DESs and BMSs) for the first time in 2016 were included in the study population. Propensity-score matching was applied to increase the comparability of the DES and BMS groups. We used a Cox proportional hazard regression analysis to compare the effectiveness of DESs and BMSs in preventing myocardial infarction, ischemic stroke, and all-cause mortality. A subgroup analysis was also performed. **Results:** In total, 21,608 cases were included in this study. Overall, the risk of myocardial infarction (aHR = 0.82; 95% CI: 0.78–0.85), ischemic stroke (aHR = 0.88; 95% CI: 0.81–0.95), and mortality (aHR = 0.61; 95% CI: 0.57–0.65) in the DES group were significantly lower than those in the BMS group. However, in some special cases, the results were not statistically significant. In particular, in patients with obesity (aHR = 2.61; 95% CI: 1.20–5.69), the DES group appeared to have a significantly higher long-term intermediate ischemic risk than the BMS group. **Conclusions and Relevance:** In conclusion, although DESs were more effective than BMSs in reducing the risk of long-term myocardial infarction, ischemic stroke, and mortality, this study also found that, in some cases, the advantages of DESs over BMSs were not clearly observed.

## 1. Introduction

A percutaneous coronary intervention (PCI, formerly known as an angioplasty with a stent) is one of the main treatments for coronary artery disease (CAD). The coronary stents for patients with stable ischemic heart disease that are currently in clinical use are divided into traditional bare-metal stents (BMSs) and drug-eluting stents (DESs) [1,2]. Traditional bare-metal stents include cobalt alloys, stainless steel, and other materials. The recoverage rate of vascular endothelium can reach 90% three to six months after placement, which can reduce the probability of coronary thrombosis. According to the guidelines issued by the American College of Cardiology and the American Heart Association, dual antiplatelet therapy should be taken for at least one month after surgery. However, within six months, about 20–30% of patients still have vascular restenosis due to the improper proliferation of vascular intimal cells [2,3]. A DES is a BMS coated with a drug polymer that inhibits the proliferation of the intimal cells, which can effectively reduce the probability of postoperative vascular restenosis. In patients undergoing PCI, DESs should be used rather than BMSs to prevent restenosis, myocardial infarction (MI), or acute stent thrombosis [2,4,5].

Several clinical trials and meta-analyses have confirmed that the clinical efficacy and prognosis, such as the vascular restenosis rate, MIs, and mortality, of patients undergoing DESs are better than those of patients undergoing BMSs [4,6,7]. Previous studies compared the prognosis and safety of DESs and BMSs and found that the incidence of major adverse cardiac events (MACE), acute MIs, and mortality were significantly lower in DESs than in BMSs [8,9]. In the past, some scholars further found that the incidence of MIs and death with DES use in the first year after placement was significantly lower than that in BMS, but there was no difference in the second and third years [4,10]. However, previous studies also pointed out that the risk of ischemic stroke with DES use is 1.02 times that with BMS use, but this is not statistically significant [11]. Although the overall advantages of DESs may be greater than those of BMSs, the price of DESs is higher than that of BMSs. The insurance system in many countries can only cover BMSs, and patients need to pay for DESs, which is expensive in full or in part. Therefore, the BMS market still exists today, especially for patients with weak economic conditions.

A significant effort has been made to compare DESs and BMSs with respect to reducing the risk of MIs and mortality following the procedure of cardiac stent placement. What seems to be lacking, however, is a full understanding of the advantages of DESs over BMSs in preventing the long-term risk of MIs and cardiac mortality in various contextual populations. This study aimed to address this gap. Furthermore, the heart–brain axis is a recent research topic in cardiac medicine. It includes the interdependence of the two-way blood circulation between the heart and the brain [12]. The type of stent may not only affect the results of the procedure in the short term, but it may also cause outcomes such as cerebral vascular embolism (ischemic stroke) or overall mortality in the long term. Previous studies evaluated the difference in the ischemic stroke risk between these two stent populations, but most analyzed the stroke risk in the early postprocedural period as a procedural complication. This study aimed to analyze the overall benefit in the reduction in the ischemic stroke risk between patients with DESs and BMSs even beyond the periprocedural period. We also further comprehensively analyzed the advantages of DESs relative to BMSs in avoiding the long-term risk of ischemic stroke in various populations.

## 2. Methods

### 2.1. Data Source and Cohort Selection

This was a retrospective cohort study. We obtained our data from the National Health Insurance Research Database (NHIRD) in Taiwan from 2015 to 2019 and selected the study sample from it. Inclusion criteria were patients who underwent coronary stent placement between January and December 2016 (ICD-9 coding: 36.06) (ICD-10 coding: 027034Z, 02703DZ, 027044Z, 02704DZ, 027134Z, 02713DZ, 027144Z, 02714DZ, 027234Z, 02723DZ, 027244Z, 02724DZ, 027334Z, 02733DZ, 027344Z, 02734DZ, 0270346, 02703D6, 0270446, 02704D6, 0271346, 02713D6, 0271446, 02714D6, 0272346, 02723D6, 0272446, 02724D6, 0273346, 02733D6, 0273446, and 02734D6). In this study, the patient’s first coronary stent placement date in 2016 was used as the index date.

**Figure 1:**
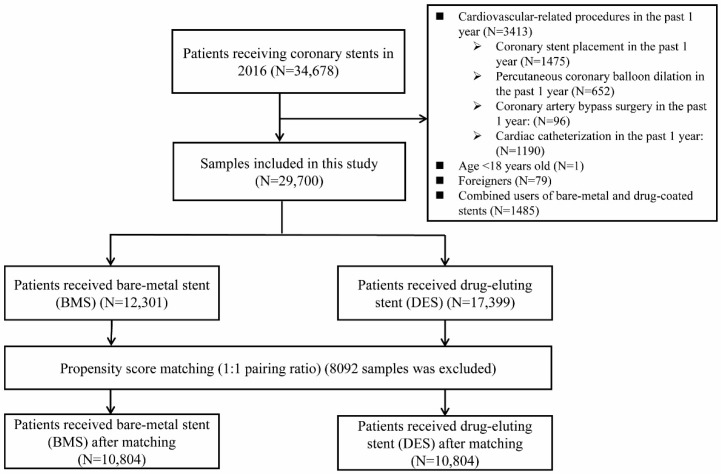
Cohort selection process.

Exclusion criteria included (1) patients who had undergone cardiovascular-related procedures in the past year (including coronary stent placement, percutaneous coronary balloon dilation, cardiovascular bypass surgery, and cardiac catheterization); (2) patients under the age of 18; (3) foreigners; and (4) patients with combined use of DESs and BMSs. The final study included samples from 29,700 patients of whom 17,399 were using DESs and of whom 12,301 were using BMSs. We then used propensity-score pairing to calculate the individual propensity for grouping patients with variables that may have influenced patients’ stent selection (demographic characteristics, disease severity, previous cardiovascular history, and number of stent placements). Next, a 1:1 matching ratio was used for sample pairing, and 21,628 people were finally included (DES: 10,814 people) (BMS: 10,814 people). The sample selection process is shown in Figure 1.

### 2.2. Outcomes

The occurrence of MI, ischemic stroke, and all-cause mortality was the outcome of this study. We used the date of the patients’ first stent placement in 2016 as the index date for all patients in order to observe whether the patients had a diagnosis of MI (ICD-9: 410 and 411) (ICD-10: I21, I22, and I24) or ischemic stroke (ICD-9: 433–434) (ICD-10: I63, I65, and I66) within three years. We used their first diagnostic record as the date of MI or ischemic stroke. For all-cause mortality, we also used the date of their first stent placement in 2016 as the index date to observe whether the patients died within three years.

### 2.3. Measurements

In this study, possible influencing factors were divided into personal characteristics and hospital features. The personal characteristics included (1) demographic features comprising gender, age, residence (northern region, central region, southern region, eastern region, and outlying islands), and monthly insurance amount; (2) diseases, including Charlson Comorbidity Index (CCI) [13], history of cardiovascular diseases (coronary artery disease, myocardial infarction, ischemic stroke, peripheral vascular disease, other cerebrovascular diseases, diabetes, and chronic kidney disease), and postoperative comorbidities (hypertension, hyperlipidemia, gout, obesity, depression, and dementia); and (3) coronary artery disease treatment, including number of stents placed, number of antiplatelet drugs, antiplatelet drug use duration (acetylsalicylic acid, clopidogrel, ticlopidine, and ticagrelor), proton pump inhibitor (PPIs), and nonsteroidal anti-inflammatory drugs (NSAIDs). Identification of the relevant drugs was based on the Anatomical Therapeutic Chemical (ATC) [14] drug classification code.

### 2.4. Statistical Approaches

The baseline characteristics of the two stent groups (DES and BMS) were compared using one-way analysis of variance (ANOVA) for continuous variables and the chi-square test for categorical variables. The Kaplan–Meier survival curve and the log-rank test were used to compare the probability of MI, ischemic-stroke incidence, and mortality among the two stent groups. Furthermore, Cox proportional hazard regression was used to estimate the hazard ratios of different variables on the outcomes, including stent groups and the baseline characteristics. Subgroup analysis was also performed to further determine the advantages of DESs over BMSs in preventing the long-term risk of MI, ischemic stroke, and mortality in various contextual populations. All data management was performed using SAS Enterprise Guide 7.1 software (SAS Institute Inc.) and IBM SPSS Statistics 20 software. The statistical significance was considered at a *p*-value of <0.05.

## 3. Results

### 3.1. Characteristics of Patients with Various Stent Types

#### 3.1.1. Personal Characteristics

Table 1 presents the distribution of the sample properties of the two stent groups after probability-score matching. The results showed that there were no significant differences in the gender ratio, age distribution, place of residence, or monthly insurance amount between the DES and BMS groups. In both the DES group and the BMS group, men accounted for 76% of the sample, while patients over the age of 65 accounted for 53%, with an average age of 65.8 years in the DES group and 66.0 years in the BMS group.

The distributions of the CCI scores for both groups were also similar. The patients with 0 ≤ CCI ≤ 1 accounted for the largest number of patients (about 60%). About 54% had a history of coronary artery disease, 13% had a history of myocardial infarction, about 8% had a history of ischemic stroke, about 2.6% had a history of peripheral vascular disease, about 6% had a history of other cerebrovascular diseases, about 40% had a history of diabetes, and about 18% had a history of chronic kidney diseases.

Regarding the postoperative comorbidities, both groups had chronic diseases such as hypertension, hyperlipidemia, and diabetes as the main comorbidities among which the rate of hypertensive patients reached 85%. There was no significant difference between the two groups. After the two groups were matched, the proportions of patients with hyperlipidemia (79.63%) in the DES group were significantly higher than those in the BMS group (72.78%). The rate of dementia (8.71%) was significantly lower in the DES group than in the BMS group (9.93%). Other comorbidities (gout, obesity, and depression) did not reach statistical significance between the two groups.

There was no difference in the number of stents placed between the two groups. The number of stents placed not only reflected the severity of the patient’s disease but also served as a reference for choosing the type of stent. Most of the patients in the two groups received one stent (around 65% in the two groups), and nearly 35% required two stents. Regarding drug utilization, 89.90% of the patients in the DES group used more than two antiplatelet drugs, which was significantly higher than their use by those in the BMS group (83.27%). Moreover, the rate of the duration of antiplatelet drug use greater than six months was significantly higher in the DES group (64.58%) than in the BMS group (57.19%). In addition, the use rate of PPIs (39.44%) in the DES group was lower than that in the BMS group (41.54%) without statistical significance, while the usage rate of NSAIDs in the DES group (74.92%) was higher than that in the BMS group (71.65%), and the difference was statistically significant.

#### 3.1.2. Kaplan–Meier Curve

**Figure 2:**
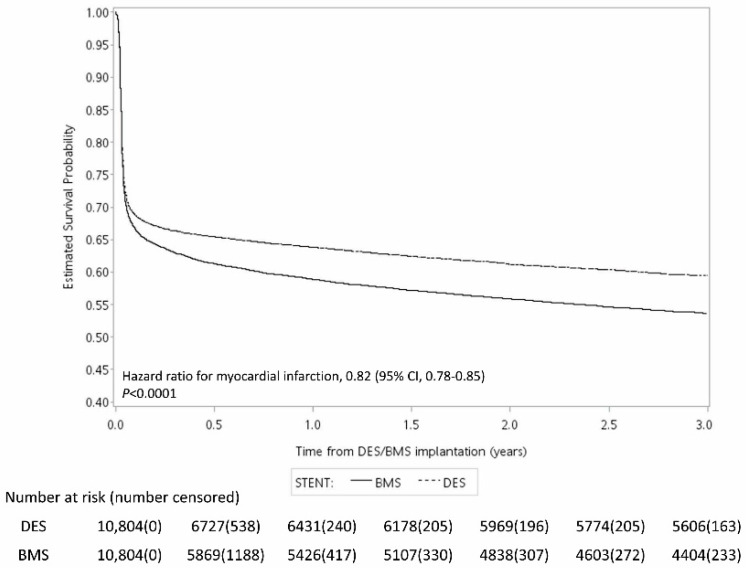
Kaplan–Meier curve for the risk of myocardial infarction within three years after surgery.

**Figure 3:**
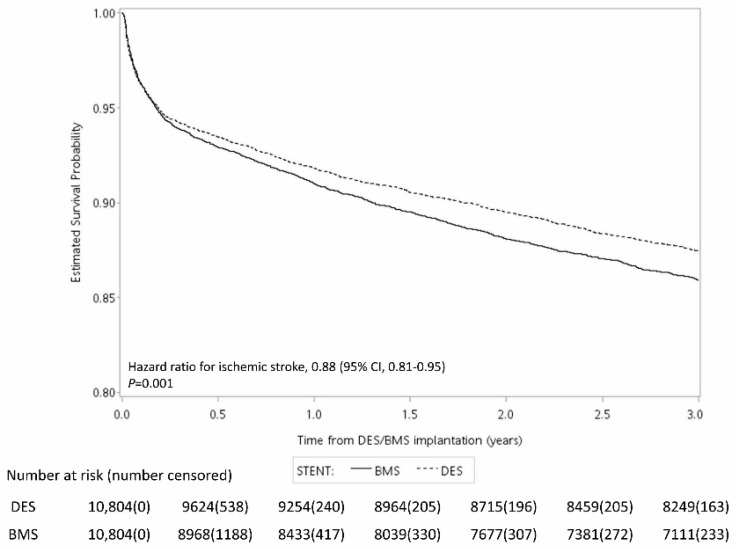
Kaplan–Meier curve for the risk of ischemic stroke within three years after surgery.

**Figure 4:**
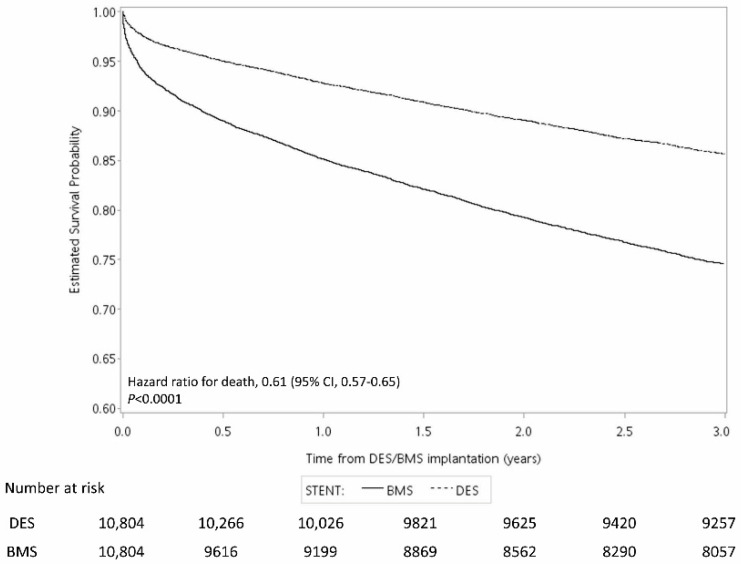
Kaplan–Meier curve for the risk of mortality within three years after surgery.

Figure 2, Figure 3 and Figure 4 show the Kaplan–Meier curve for the incidence of MI, ischemic stroke, and mortality within three years after surgery. The results showed that the risk of all three outcomes were lower in the DES group than in the BMS group. The results of the log-rank test showed that the difference between the two groups was statistically significant (Figure 2, Figure 3 and Figure 4).

#### 3.1.3. Cox Proportional Hazard Model

Table 2 shows that, after adjusting for other factors, the incidence of MI (aHR = 0.82; 95% CI: 0.78–0.85), ischemic stroke (aHR = 0.88; 95% CI: 0.81–0.95), and mortality (aHR = 0.61; 95% CI: 0.57–0.65) in the DES group was significantly lower than that in the BMS group

In addition to the type of stent, other risk factors for MI included male gender, a history of MI or chronic kidney disease, a comorbidity of hyperlipidemia, more antiplatelet drugs (>2), a longer antiplatelet drug use duration (>6 months), and the long-term use of PPIs. Other risk factors for ischemic stroke included male gender; old age (≥65); a higher CCI index score (≥2); a history of ischemic stroke, peripheral vascular diseases, other cerebrovascular diseases, or diabetes; a comorbidity of hypertension, hyperlipidemia, depression, or dementia; treatment with more than two stents; and PPI or NSAID use. Other risk factors for mortality included old age (≥65); a higher CCI index score (≥2); a history of MI, ischemic stroke, peripheral vascular diseases, diabetes, or chronic kidney disease; treatment with more than two stents; and PPI use.

The results of the subgroup analysis are summarized in Table 3. For most of the populations, DESs were more able to prevent MI than BMS. No significant differences between the two were found in the following populations: patients with a history of peripheral vascular disease or chronic kidney disease and patients with a comorbidity of obesity. Our subgroup analyses also confirmed that DESs could prevent ischemic stroke to a greater extent than BMSs in most of the populations. However, in the following populations, no significant differences between the two groups were found: females; those of an age ≥ 65; those with a CCI ≥ 2; those with a history of ischemic stroke (aHR = 1.02; 95% CI: 0.90–1.15), peripheral vascular disease, other cerebrovascular diseases (aHR = 1.00; 95% CI: 0.83–1.21), or chronic kidney disease; patients with a comorbidity of depression or dementia; those with lower antiplatelet drug use (≤1); and those undergoing the long-term use of PPIs. However, in patients with obesity (aHR = 2.61; 95% CI: 1.20–5.69), the DES group appeared to have a significantly higher long-term intermediate ischemic risk than the BMS group. The findings also found that DESs were associated with a significantly lower risk of mortality than BMSs in most populations. However, this result was not only found in patients with obesity (Table 3).

## 4. Discussion

Some previous studies [4,5,10] have shown a significant advantage of DESs in the prevention of MI compared to BMSs, while, in others [11], no statistically significant difference was found between the two groups. Using a larger sample size, this study further confirmed the benefits of DESs compared to BMSs in the prevention of future long-term MI in most of the populations. In a few populations, the difference between the two groups was not obvious. The reason for the statistically insignificant results may be that the sample size of these populations was not sufficiently large. This result may explain why some previous studies [11] did not clearly show the advantage of DESs over BMSs in preventing the risk of MI.

A stroke is a known complication after myocardial infarction (MI). Parts of the causes of ischemic stroke and MI are similar usually because the blood coagulates into a “thrombus” and blocks the blood vessels in different parts of the body, forming an unhealthy state. With respect to the causes of the blood clotting into a thrombus, in addition to common causes such as hyperlipidemia, diabetes, high blood pressure, obesity, stress [15], or the patient’s blood being prone to clotting [16], etc., a thrombus being generated during vascular reconstruction may be the main reason [15,16,17]. We could reasonably infer that DESs had more coatings of the drug polymer that inhibits the proliferation of the intrinsic cells than BMSs did, which could effectively reduce the probability of postoperative vascular restenosis and thrombus formation, thereby reducing the subsequent risk of ischemic stroke caused by a thrombosis blocking the cerebral blood vessels. Despite this, a previous study [11] used a smaller number of cases (*n* = 3051) and indicated that the risk of ischemic stroke was not significantly different between DES and BMS patients (aHR = 1.02; 95% CI: 0.67–1.53; *p*-value = 0.937). However, the current study used a larger sample size (*n* = 21,628) and found that the patients who received DESs had a significantly lower risk of ischemic stroke (aHR = 0.89; 95% CI: 0.82–0.96; *p*-value = 0.0027) than those who used BMSs did. 

In addition, the results of our subgroup analyses also showed that the risk of ischemic stroke was significantly lower in the DES group compared with the BMS group in parts of some populations. For the other populations, the risk of ischemic stroke was similar between the two groups. These results may explain why past studies [11] did not show a significant difference between the DES and BMS groups in terms of the incidence of ischemic stroke. Surprisingly, however, the results of this study showed that the DES group had a higher risk of subsequent ischemic stroke than the BMS group. The small number of patients with obesity in the cases included in this study (DES: 125, 1.16%) (BMS: 24, 1.15%) may account for this result.

The results of this study also found that in nonobese patients, compared with the BMS group, the DES group had a significantly lower risk of ischemic stroke. However, in obese patients, the DES group was not better than the BMS group in preventing MI, ischemic stroke, and death, and, especially for ischemic stroke, the result was the opposite. We speculated that the possible reason for this was that the number of obese patients included in this study was small or because obese patients are more likely to be complicated with complex mixed diseases, such as hyperlipidemia, diabetes, hypertension, heart disease, and other systemic metabolisms [18,19], and these complex diseases may have increased the risk of stroke due to cerebrovascular stenosis or a thrombus blockage. [20,21] As for the direct or indirect impact mechanism of the differences in the types of cardiac stents and their ischemic stroke outcomes, further research is needed for clarification.

Many cardiac disease and stroke patients and people seeking to avoid these events are treated with two types of antiplatelet agents to prevent blood clotting, or so called dual antiplatelet therapy (DAPT) [22,23,24]. This study found that, for patients who used more than two antiplatelet drugs after cardiac stenting, DESs were more effective than BMSs in preventing MI and ischemic stroke. However, for patients who only used zero or one antiplatelet drug, DESs did not have a significant advantage over BMSs in the prevention of MI and ischemic stroke. According to this result, DES users were suggested to use more than two kinds of antiplatelet drugs in order to continue to have a significant effect on the prevention of MI or ischemic stroke.

Several previous meta-analysis results showed that all-cause mortality was not significantly different between DES and BMS patients [4,5]. Overall, a significant advantage of DESs over BMSs in preventing all-cause mortality (aHR = 0.64; 95% CI: 0.60–0.68) was observed in this study, which was similar to the results of many previous studies [10,11]. To find out why the results of the meta-analyses were not significant, this study further used subgroup analyses and found that the DES group had a significantly lower risk of mortality than the BMS group in most cases, except for in patients with obesity. The possible reason was that the obese patients had a higher risk of death, and the mortality factors of the obese patients were more complicated [18,19]. The type of cardiac stent was only one of multiple influencing factors for the risk of death, and its influence may not be important enough.

The results of this study showed that the mortality in the DES group was around 14% and that that in the BMS group was around 25% at 3 years. Previous studies on 3-year mortality after PCI have had widely varying results (3.6%–25%) [25,26,27,28,29,30,31]. The difference in the mortality rate could reflect a health care different from most countries in the world. Usually, real-world patients may be less healthy on average than clinical trial admissions. In addition, there is heterogeneity in the populations selected in studies, such as the sex ratio of patients, average age, severity of heart disease, overall health status, PCI treatment process and method, follow-up care mode, etc., all of which affect the mortality rate.

This study had several limitations. First, because the database used in this study was limited with regard to other socioeconomic status information (such as education level, job type, and economic status) and other personal factors that may have affected the outcome (such as height, weight, and BMI), it was impossible to understand the relationship between these factors and the risk of the outcomes, which needs to be explored further in the future. Second, the claims database used in this study did not include the results of instrumental inspections, such as ejection fraction, so this variable was not considered. To clearly define the included patients’ condition of heart disease, including stable angina or acute coronary syndrome, this study adopted a new user design which included all the patients who underwent coronary stent placement and excluded the patients who had undergone cardiovascular-related procedures in the past year (including coronary stent placement, percutaneous coronary balloon dilation, cardiovascular bypass surgery, and cardiac catheterization). Also, their history of cardiovascular diseases (including coronary artery disease, myocardial infarction, ischemic stroke, peripheral vascular disease, and other cerebrovascular diseases) was considered during matching. To clearly define the condition of treatment for each patient, this study used the number of antiplatelet drugs, antiplatelet drug use duration, and number of stents placed as confounding variables during adjustment. Third, this study could not confirm the patients’ behavior (such as compliance, smoking, diet, exercise, etc.) after stent treatment; therefore, the inferences from the study results were limited. Finally, this study used all-cause mortality instead of cardiac mortality as the outcome because the database used in this study did not include causes of death. Nevertheless, since this study focused on comparing the long-term risk differences between the two stents, all-cause mortality was a better proxy for the overall mortality risk.

## 5. Conclusions

Overall, this study used big data to confirm the long-term advantages of DESs over BMSs in preventing the risk of myocardial infarction, ischemic stroke, and overall mortality. Furthermore, our results further highlighted the differences between the two stents in various populations. In most cases, DESs obviously had greater benefits than BMSs; however, in a few cases, the advantages of DESs were not significant. Especially in the obese patients, the question of whether DESs were still superior to BMSs in reducing the long-term incidence of ischemic stroke remains to be confirmed by further and larger data studies. DES use combined with more than two types of antiplatelet drugs could have a more significant effect on the prevention of MI and ischemic stroke than BMS use.

## Figures and Tables

**Table 1:** Baseline characteristics of patients with coronary stent placement.

Variables	DES Group		BMS Group		
	(*n* = 10,804)		(*n* = 10,804)		*p*-Value
	Number	%	Number	%	
**Gender**					0.8489
Male	8190	75.81	8178	75.69	
Female	2614	24.19	2626	24.31	
**Age (years old)**	Mean = 65.8		Mean = 66.0		0.9132
18 ≤ Age < 65	5106	47.26	5114	47.33	
≥65	5698	52.74	5690	52.67	
**Charlson Comorbidity Index (CCI) score**					0.2006
0 ≤ CCI ≤ 1	6575	60.86	6483	60.01	
≥2	4229	39.14	4321	39.99	
**History of cardiovascular diseases and other diseases**					
Coronary artery disease	5802	53.70	5880	54.42	0.2870
Myocardial infarction	1450	13.42	1483	13.73	0.5122
Ischemic stroke	908	8.40	906	8.39	0.9609
Peripheral vascular disease	279	2.58	283	2.62	0.8642
Other cerebrovascular diseases	698	6.46	699	6.47	0.9779
Diabetes	4270	39.52	4296	39.76	0.7177
Chronic kidney disease	1946	18.01	1977	18.3	0.5843
**Postoperative comorbidities**					
Hypertension	9169	84.87	9194	85.10	0.6340
Hyperlipidemia	8603	79.63	7863	72.78	<0.0001 ***
Gout	2443	22.61	2342	21.68	0.0980
Obesity	123	1.14	124	1.15	0.9490
Depression	564	5.22	566	5.24	0.9513
Dementia	941	8.71	1073	9.93	0.002 **
**Number of stents placed**					0.5676
1	7072	65.46	7032	65.09	
≥ 2	3732	34.54	3772	34.91	
**Number of antiplatelet drugs**					<0.0001 ***
0 ≤ number ≤ 1	1091	10.10	1808	16.73	
≥2	9713	89.90	8996	83.27	
**Antiplatelet drug use duration**	Mean = 217.0		Mean = 193.4		<0.0001 ***
<6 months	3827	35.42	4625	42.81	
≥6 months	6977	64.58	6179	57.19	
**Proton pump inhibitor (PPI)**	4348	39.44	4488	41.54	0.0527
**NSAID**	8094	74.92	7741	71.65	<0.0001 ***

DES = drug-eluting stent; BMS = bare-metal stent; ** *p* < 0.01; and *** *p* < 0.001.

**Table 2:** Results of the Cox proportional hazard model.

Variables	Myocardial Infarction			Ischemic Stroke			Mortality		
	aHR	95% CI	*p*-Value	aHR	95% CI	*p*-Value	aHR	95% CI	*p*-Value
**Type of stent, DES**	0.82	0.78–0.85	<0.0001 ***	0.88	0.81–0.95	0.001 **	0.61	0.57–0.65	<0.0001 ***
**Gender, male**	1.12	1.07–1.19	<0.0001 ***	1.11	1.01–1.21	0.0275 *	0.91	0.86–0.98	0.0081 **
**Age, ≥ 65**	0.87	0.84–0.91	<0.0001 ***	1.48	1.35–1.62	<0.0001 ***	2.26	2.09–2.43	<0.0001 ***
**Charlson Comorbidity Index (CCI) score,** **≥ 2**	0.92	0.87–0.97	0.0027 **	1.27	1.16–1.4	<0.0001 ***	1.54	1.42–1.67	<0.0001 ***
**History of cardiovascular diseases and others**									
**Coronary artery disease**	0.34	0.32–0.35	<0.0001 ***	1.03	0.95–1.11	0.5481	0.90	0.85–0.96	0.0016 **
**Myocardial infarction**	2.57	2.44–2.7	<0.0001 ***	0.88	0.79–0.99	0.031 *	1.07	0.98–1.16	0.1159
**Ischemic stroke**	0.86	0.79–0.94	0.0008 ***	11.53	10.56–12.59	< 0.0001 ***	1.19	1.08–1.3	0.0003 ***
**Peripheral vascular disease**	1.02	0.88–1.18	0.7968	1.40	1.16–1.68	0.0004 ***	1.55	1.37–1.75	<0.0001 ***
**Other cerebrovascular diseases**	0.93	0.84–1.02	0.1142	1.13	1.02–1.26	0.024 *	1.05	0.94–1.17	0.3554
**Diabetes**	0.90	0.85–0.94	<0.0001 ***	1.09	1–1.19	0.0455 *	1.30	1.21–1.39	<0.0001 ***
**Chronic kidney disease**	1.08	1.01–1.16	0.0214 *	0.90	0.81–0.99	0.0361*	1.49	1.38–1.61	<0.0001 ***
**Postoperative comorbidities**									
**Hypertension**	0.85	0.8–0.9	<0.0001 ***	1.80	1.51–2.15	< 0.0001 ***	0.71	0.65–0.77	<0.0001 ***
**Hyperlipidemia**	1.14	1.08–1.2	<0.0001 ***	1.22	1.11–1.35	< 0.0001 ***	0.41	0.39–0.44	<0.0001 ***
**Gout**	0.96	0.91–1.01	0.1335	1.07	0.98–1.17	0.1574	0.88	0.81–0.95	0.0012 **
**Obesity**	0.81	0.66–1	0.0481 *	1.21	0.87–1.67	0.2617	0.71	0.48–1.07	0.0998
**Depression**	0.92	0.83–1.01	0.0832	1.19	1.04–1.36	0.0139 *	0.87	0.76–1	0.0572
**Dementia**	1.04	0.97–1.13	0.2754	1.93	1.75–2.13	<0.0001 ***	1.07	0.98–1.17	0.1382
**Number of stents placed, ≧ 2**	0.90	0.86–0.94	<0.0001 ***	1.15	1.06–1.24	0.0005 ***	1.18	1.11–1.26	<0.0001 ***
**Number of antiplatelet drugs, ≥ 2**	1.58	1.46–1.71	<0.0001 ***	1.04	0.92–1.17	0.5747	0.35	0.33–0.38	<0.0001 ***
**Antiplatelet drug use duration, ≥ 6 months**	1.10	1.05–1.15	<0.0001 ***	0.93	0.86–1.01	0.0727	0.38	0.36–0.41	<0.0001 ***
**Proton pump inhibitor (PPI)**	1.08	1.04–1.13	0.0004 ***	1.32	1.22–1.42	<0.0001 ***	1.45	1.36–1.54	<0.0001 ***
**NSAID**	0.95	0.9–0.99	0.0264 *	1.12	1.02–1.24	0.0215 *	0.39	0.37–0.42	<0.0001 ***

* *p* < 0.05, ** *p* < 0.01, and *** *p* < 0.001.

**Table 3:** Results of the Cox proportional hazard model with subgroup of DES vs. BMS (ref.).

Variables	Myocardial Infarction			Ischemic Stroke			Mortality		
	aHR	95% CI	*p*-Value	aHR	95% CI	*p*-Value	aHR	95% CI	*p*-Value
**Gender**									
Male	0.81	0.77–0.85	<0.0001 ***	0.85	0.77–0.93	0.0003 ***	0.60	0.55–0.64	<0.0001 ***
Female	0.86	0.78–0.94	0.001 **	0.99	0.86–1.15	0.9042	0.62	0.56–0.69	<0.0001 ***
**Age (years old)**									
18 ≤ Age < 65	0.83	0.78–0.88	<0.0001 ***	0.78	0.68–0.90	0.0005 ***	0.49	0.43–0.56	<0.0001 ***
≥65	0.80	0.75–0.85	<0.0001 ***	0.94	0.85–1.03	0.1637	0.65	0.61–0.70	<0.0001 ***
**Charlson Comorbidity Index (CCI) score**									
0 ≤ CCI ≤ 1	0.81	0.77–0.86	<0.0001 ***	0.81	0.72–0.91	0.0003 ***	0.57	0.51–0.63	<0.0001 ***
≥2	0.84	0.79–0.90	<0.0001 ***	0.95	0.85–1.05	0.2916	0.65	0.60–0.70	<0.0001 ***
**History of cardiovascular diseases and others**									
Coronary artery disease									
Y	0.75	0.70–0.80	<0.0001 ***	0.84	0.76–0.93	0.0005 ***	0.59	0.54–0.64	<0.0001 ***
N	0.88	0.84–0.93	<0.0001 ***	0.94	0.83–1.06	0.3284	0.64	0.58–0.70	<0.0001 ***
Myocardial infarction									
Y	0.89	0.81–0.97	0.0119 *	0.70	0.57–0.87	0.0013 **	0.63	0.54–0.73	<0.0001 ***
N	0.81	0.77–0.85	<0.0001 ***	0.91	0.84–0.99	0.0249 *	0.60	0.56–0.65	<0.0001 ***
Ischemic stroke									
Y	0.72	0.61–0.85	<0.0001 ***	1.02	0.90–1.15	0.7910	0.63	0.53–0.75	<0.0001 ***
N	0.82	0.79–0.86	<0.0001 ***	0.81	0.73–0.90	<0.0001 ***	0.61	0.57–0.65	<0.0001 ***
**Peripheral vascular disease**									
Y	0.90	0.68–1.20	0.4812	0.95	0.66–1.38	0.7929	0.77	0.60–0.98	0.0314 *
N	0.82	0.78–0.85	<0.0001 ***	0.88	0.81–0.95	0.0010 **	0.60	0.56–0.64	<0.0001 ***
Other cerebrovascular diseases									
Y	0.83	0.69–0.99	0.0483 *	1.00	0.83–1.21	0.9986	0.62	0.50–0.77	<0.0001 ***
N	0.82	0.78–0.85	<0.0001 ***	0.85	0.78–0.93	0.0002 ***	0.61	0.57–0.65	<0.0001 ***
Diabetes									
Y	0.82	0.77–0.88	<0.0001 ***	0.90	0.80–1.00	0.0494 *	0.62	0.57–0.67	<0.0001 ***
N	0.82	0.78–0.86	<0.0001 ***	0.86	0.77–0.96	0.0079 **	0.60	0.55–0.66	<0.0001 ***
Chronic kidney disease									
Y	0.92	0.83–1.02	0.1034	0.99	0.85–1.16	0.8919	0.73	0.66–0.80	<0.0001 ***
N	0.80	0.77–0.84	<0.0001 ***	0.85	0.78–0.93	0.0005 ***	0.55	0.51–0.60	<0.0001 ***
Postoperative comorbidities									
Hypertension									
Y	0.82	0.78–0.86	<0.0001 ***	0.87	0.81–0.94	0.0006 ***	0.62	0.58–0.67	<0.0001 ***
N	0.83	0.75–0.92	0.0002 ***	1.04	0.73–1.47	0.8362	0.57	0.48–0.68	<0.0001 ***
Hyperlipidemia									
Y	0.81	0.78–0.85	<0.0001 ***	0.88	0.81–0.96	0.0033 **	0.56	0.52–0.61	<0.0001 ***
N	0.85	0.77–0.93	0.0005 ***	0.89	0.75–1.06	0.1950	0.68	0.62–0.75	<0.0001 ***
Gout									
Y	0.83	0.76–0.90	<0.0001 ***	0.85	0.73–0.99	0.0406 *	0.66	0.57–0.77	<0.0001 ***
N	0.82	0.78–0.86	<0.0001 ***	0.89	0.81–0.97	0.0070 **	0.60	0.56–0.64	<0.0001 ***
Obesity									
Y	0.65	0.41–1.01	0.0570	2.61	1.20–5.69	0.0161 *	0.39	0.15–1.01	0.0526
N	0.82	0.79–0.85	<0.0001 ***	0.87	0.80–0.94	0.0003 ***	0.61	0.57–0.65	<0.0001 ***
Depression									
Y	0.80	0.66–0.97	0.0253 *	0.95	0.73–1.24	0.7081	0.49	0.37–0.65	<0.0001 ***
N	0.82	0.78–0.85	<0.0001 ***	0.88	0.81–0.95	0.0014 **	0.62	0.58–0.66	<0.0001 ***
Dementia									
Y	0.75	0.65–0.86	<0.0001 ***	0.90	0.77–1.05	0.1773	0.53	0.45–0.62	<0.0001 ***
N	0.82	0.79–0.86	<0.0001 ***	0.89	0.82–0.98	0.0123 *	0.63	0.59–0.68	<0.0001 ***
**Number of stents placed**									
1	0.85	0.81–0.90	<0.0001 ***	0.85	0.77–0.94	0.0011 **	0.64	0.59–0.70	<0.0001 ***
≥2	0.74	0.69–0.80	<0.0001 ***	0.94	0.83–1.06	0.3092	0.56	0.51–0.62	<0.0001 ***
**Number of antiplatelet drugs**									
0 ≤ number ≤ 1	0.88	0.76–1.03	0.1177	0.95	0.76–1.19	0.6495	0.75	0.67–0.83	<0.0001 ***
≥2	0.81	0.78–0.85	<0.0001 ***	0.88	0.81–0.96	0.0023 **	0.58	0.53–0.62	<0.0001 ***
**Antiplatelet drug use duration**									
<6 months	0.86	0.80–0.92	<0.0001 ***	0.81	0.71–0.92	0.0012 **	0.63	0.58–0.68	<0.0001 ***
≥6 months	0.80	0.76–0.84	<0.0001 ***	0.92	0.84–1.02	0.1004	0.61	0.55–0.68	<0.0001 ***
**Proton pump inhibitor (PPI)**									
Y	0.77	0.72–0.82	<0.0001 ***	0.92	0.83–1.02	0.1043	0.64	0.59–0.69	<0.0001 ***
N	0.86	0.81–0.90	<0.0001 ***	0.84	0.75–0.94	0.0018 **	0.58	0.53–0.64	<0.0001 ***
**NSAID**									
Y	0.84	0.80–0.88	<0.0001 ***	0.89	0.81–0.97	0.0059 **	0.62	0.57–0.67	<0.0001 ***
N	0.76	0.70–0.83	<0.0001 ***	0.86	0.72–1.02	0.0869	0.64	0.58–0.70	<0.0001 ***

* *p* < 0.05, ** *p* < 0.01, and *** *p* < 0.001.

## Data Availability

The study did not report any data.

## Abbreviations

PCI represents percutaneous coronary intervention; CAD represents coronary artery diseases; BMS represents bare-metal stents; DES represents drug-eluting stents; MACE represents major adverse cardiac events; NHIRD represents National Health Insurance Research Database; CCI represents Charlson Comorbidity Index; PPI represents proton pump inhibitor; NSAIDs represents nonsteroidal anti-inflammatory drugs; and ATC represents anatomical therapeutic chemical.
